# Supplementary material for: A New Strategy for the Synthesis of Hydroxyl Terminated Polystyrene-b-Polybutadiene-b-Polystyrene Triblock Copolymer with High Cis-1, 4 Content
Source: Polymers (Basel). 2019 Apr 2;11(4):598. doi: 10.3390/polym11040598 (PMC6523885; doi:10.3390/polym11040598)
Supplement: Supplementary file 1 [file polymers-11-00598-s001.pdf]

Supplementary Materials: A New Strategy for the Synthesis of Hydroxyl Terminated Polystyrene-*b*-Polybutadiene-*b*-Polystyrene Triblock Copolymer with High Cis-1, 4 Content

Xin Min and **Xiaodong Fan**

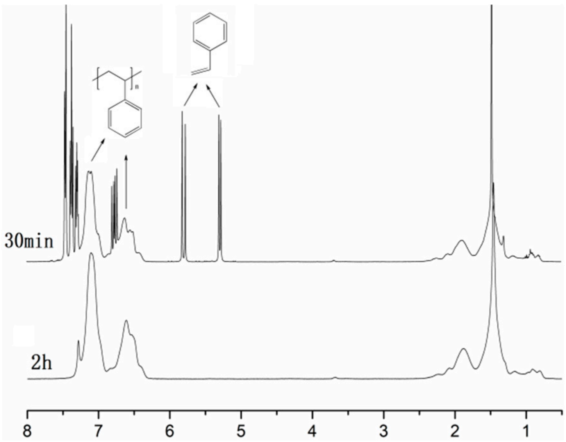

Figure S1. the ^{1}H-NMR of St polymerization process with different polymerization time.

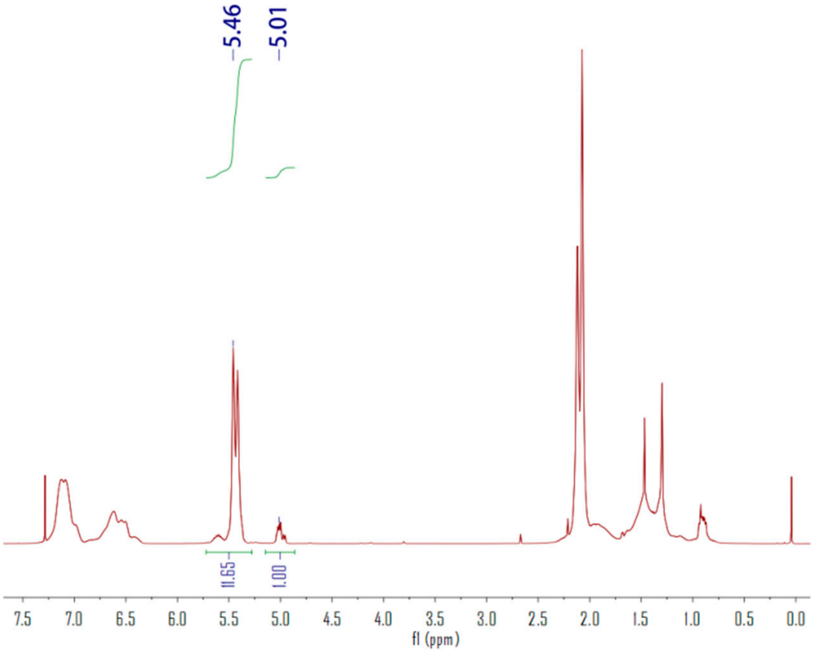

Figure S2. ^{1}H-NMR of Li/HO-SBS-OH.

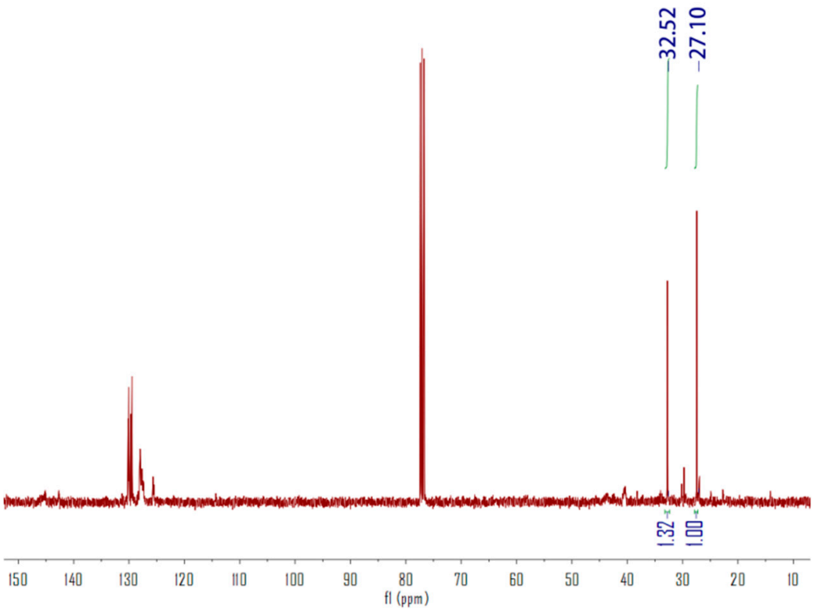

Figure S3. ^{13}C-NMR of Li/HO-SBS-OH.

The ratio of butadiene to OH can be calculated by ^{1}H-NMR which is shown in figure S4 (Initiator:St:Bd:St (molar ratio)= 0.02:1:4:1). The ratio can be calculated by formula [(a+b)+2c/3]/d, so the ratio of butadiene to OH is (392.5+2*12.7/3)/4=100.25.

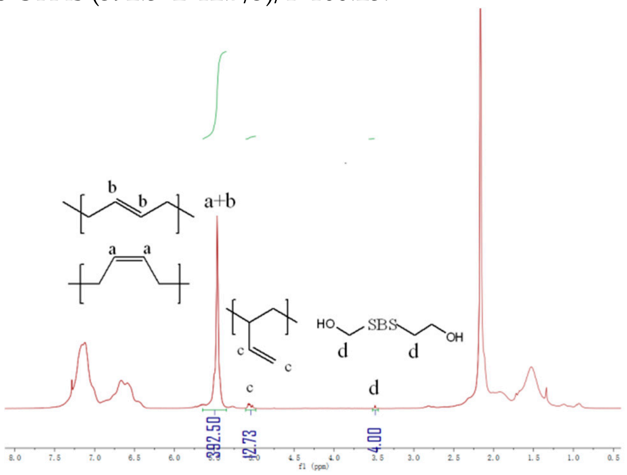

Figure S4. ^{1}H-NMR integral data of HO-SBS-OH.

Due to the effect of $CDCl_3$, ^{1}H-NMR is inappropriate to analyze the ratio of butadiene to styrene. So ^{13}C-NMR is used to calculate the ratio of butadiene to styrene which is shown in figure S5. Due to the 1,2 content is very low, thus, for convenience, the effect of 1,2 content is ignored. Then the ratio of butadiene to styrene can be estimated by formula (a+b)/2c. So the ratio of butadiene to styrene is (0.23+3.79)/2=2.01. The ratio of styrene to butadiene to OH is about (100.25/2.01):100.25:1=99.8: 200.5: 2, which mean that the degree of PS and degree of PBd is 100 and 201 respectively. So the expected M_n is $M_{nPS}+M_{nPBd}+M_{nOH}$= 100*104+201*54+2*17=21352g*mol^{-1}.

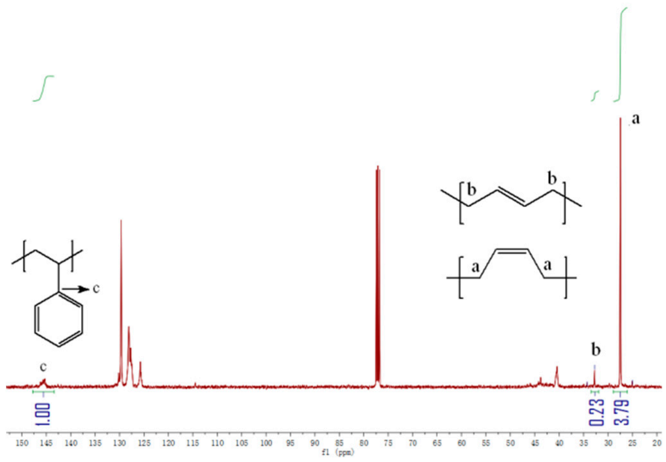

Figure S5. ^{13}C-NMR integral data of HO-SBS-OH.
